# Optimal Design Based on Closed-Loop Fusion for Velocity Bandwidth Expansion of Optical Target Tracking System

**DOI:** 10.3390/s19010133

**Published:** 2019-01-02

**Authors:** Yao Mao, Wei Ren, Yong Luo, Zhijun Li

**Affiliations:** 1 Key Laboratory of Optical Engineering, Chinese Academy of Sciences, Chengdu 610209, China; renwei9327@163.com (W.R.); ly250047087@126.com (Y.L.); zhijunhome1984@163.com (Z.L.); 2 Institute of Optics and Electronics, Chinese Academy of Science, Chengdu 610209, China; 3 Chinese Academy of Science, Beijing 100039, China

**Keywords:** MEMS gyro, optical target tracking, closed-loop fusion, optimal design

## Abstract

Micro-electro-mechanical system (MEMS) gyro is one of the extensively used inertia sensors in the field of optical target tracking (OTT). However, velocity closed-loop bandwidth of the OTT system is limited due to the resonance and measurement range issues of MEMS gyro. In this paper, the generalized sensor fusion framework, named the closed-loop fusion (CLF), is analyzed, and the optimal design principle of filter is proposed in detail in order to improve measurement of the bandwidth of MEMS gyro by integrating information of MEMS accelerometers. The fusion error optimization problem, which is the core issue of fusion design, can be solved better through the feedback compensation law of CLF framework and fusion filter optimal design. Differently from conventional methods, the fusion filter of CLF can be simply and accurately designed, and the determination of superposition of fusion information can also be effectively avoided. To show the validity of the proposed method, both sensor fusion simulations and closed-loop experiments of optical target tracking system have yielded excellent results.

## 1. Introduction

The attitude measurement technology of the optical target tracking (OTT) system, which is key to achieving high-precision laser control, has been extensively studied [1,2,3]. Gyros can sense angular jitter and provide real-time inertial attitude information about moving objects. Especially, micro-electro-mechanical system (MEMS) gyro is widely used in various practical application due to the advantages of low price, low power, and small size. Stable performance of the OTT platform is mainly limited to mechanical resonance of platform and resonance of MEMS gyro [4]. Due to the existing resonance of MEMS gyro, even if the notch filter is used to compensate the resonance, viable bandwidth of MEMS gyro is also reduced. Thus, it does not have enough ability to accurately realize high-bandwidth control.

When the stiffness of the platform is improved, and the mechanical resonance position of the system is improved, the measurement range and resonance of MEMS gyro become the main factors limiting the stability control bandwidth of the OTT. Tang realized, double closed-loop control is based on accelerometer with wide bandwidth [5], but the acceleration is proportional to the signal frequency, and the proportional factor of the wide bandwidth accelerometer is bound to be smaller. For low frequency and small amplitude vibration, the output signal is almost obliterated in the noise. It is necessary to improve the drift correction ability of position loop, otherwise the closed-loop control of the system cannot be achieved [6,7].

A solution to the resonance and detection range limitations of gyro is to adopt a multi-sensor fusion technology [8,9,10]. The ability of the sensor to detect frequencies can be easily divided into low-bandwidth measurement sensors and high-bandwidth measurement sensors [11,12]. Therefore, sensor data can be combined into two different characteristics in the form of a combining filter. This method is very simple to use, but the frequency characteristics of the relevant sensor must be known or measured. Otherwise, the combining filter cannot be designed to eliminate the problem of overlapping frequency response during the fusion process [13]. Therefore, the application of this method is limited to scenarios known to the sensor model. In addition, in the field of rigid body angular motion measurements and mobile-robot attitude estimation, a Kalman filter can be utilized to estimate the amount of low frequency error in the data collected by the high-bandwidth sensor. However, high-order time-variant Kalman filters are needed in the absence of a high-bandwidth sensor system model, which is difficult to implement [14,15,16].

To overcome the shortcomings of the aforementioned methods, Algrain proposed an alternative method called closed-loop fusion (CLF) [17]. In this method, the measurement data of the low-bandwidth sensor and the high-bandwidth sensor are adjusted by a closed-loop filter. Compared to the above method, it does not require an accurate model or transfer function of the sensor, and the feedback compensation structure can effectively eliminate the drift error. However, he did not point out a reasonable design method for the closed-loop filter. Finding a classical filter that satisfies minimum fusion error criteria is a time-consuming procedure. During the experiment, we found that if the closed-loop bandwidth of corrector is too low, the high-bandwidth sensor cannot track the low-bandwidth sensor. Conversely, if the closed-loop bandwidth of corrector is too high, the high frequency will affect the low frequency correction term and invalidate the correction. With this in mind, we proposed an optimized design guide for CLF filters. Finally, we utilized the proposed design guidelines to achieve higher velocity bandwidth expansion of OTT and improve the robust stability of the system.

In the second part, we analyze the basic principles of fusion and derive the optimal design of the CLF filter. In the third part, the simulation and velocity closed-loop control experiments verify the correctness of these design guidelines. The fourth part deals with these conclusions.

## 2. Closed-Loop Fusion Framework

In this part, we study the CLF structure and theoretically analyze the optimal fusion design implementation. Our researchers begin with a simple combination fusion principle, and then we propose our own fusion structure based on the basic principles of fusion.

### 2.1. Basic Principle of Fusion

**Figure 1:**
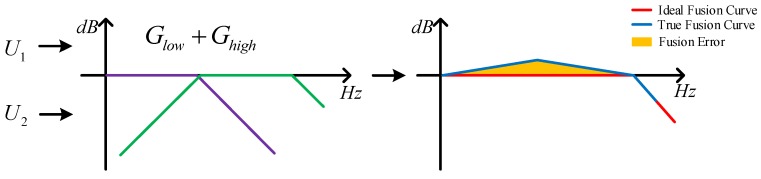
Fusion process of sensor signals in the frequency domain.

It is assumed that there are two sensors $U_{1}$ and $U_{2}$ with inconsistent characteristics, the two sensors have low-bandwidth property and high-bandwidth property, respectively, and the transfer functions can be defined as $G_{low}$ and $G_{high}$ in order to obtain a signal with all-pass characteristics over the entire spectrum. The simplest fusion idea is to directly add the two sensors data linearly. However, the simple linear addition processing operation inevitably has signal overlap in the entire frequency band. Figure 1 shows in the process of sensor fusion. The error resulted from the overlap is deviated from the information expressed by the real object.

**Figure 2:**
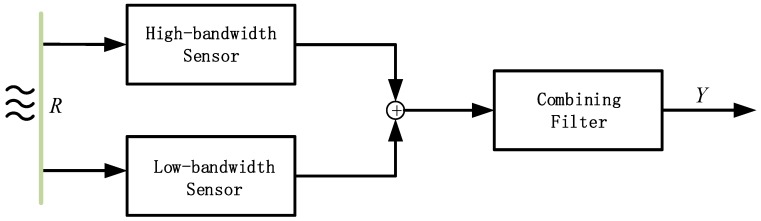
Fusion process of combining filter method.

The traditional approach is that the combining filter method is adopted to eliminate the fusion error by sensor characteristic cancellation in Figure 2. If we know the expressions of $G_{low}$ and $G_{high}$, then linear overlap errors can be removed when the combining filter is $\frac{1}{G_{low}+G_{high}}$ in theory. Note that the premise of implementing this method is the fact that we can know or measure the transfer function of the sensor.

### 2.2. Closed-Loop Fusion Scheme

**Figure 3:**
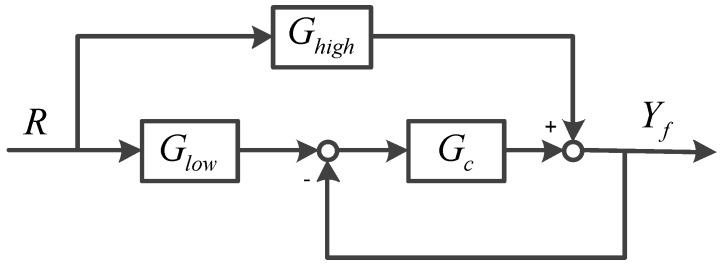
The CLF Scheme.

In this section, an advance fusion structure is proposed for fusion technologies. Its advantage is that it does not need to know the sensor property, and it conveniently realizes the optimal fusion design. The CLF network with real-time correction is shown in Figure 3. $G_{c}$ represents fusion filter which is used to correct the fusion error of two data channels in real time. R is the physical motion quantity, and $Y_{f}$ is the fusion output.

The transfer function of CLF can be expressed as

(1) $$G_{cl\_fusion}=\frac{Y_{f}}{R}=\frac{1}{1+G_{c}}\cdot G_{low}+\frac{G_{c}}{1+G_{c}}\cdot G_{high}$$

From the perspective of control, according to the transfer function of CLF, $\frac{1}{1+G_{c}}$ can be regarded as the system tracking performance to input. $\frac{G_{c}}{1+G_{c}}$ represents the system’s ability to suppress disturbances. It is characterized by the ability to track low-bandwidth sensor signal at low frequencies and highlight high-bandwidth sensor signals at high frequencies. Therefore, Equation (1) can be rewritten as the following form

(2) $$G_{cl\_fusion}=G_{close}\cdot G_{low}+G_{inhibit}\cdot G_{high}$$

$G_{close}$ and $G_{inhibit}$ represent the tracking and suppression performance of the CLF network structure, respectively.

### 2.3. Closed-Loop Fusion Design

In order to obtain the desired fusion performance, the following two rules should be followed when we design the fusion filter $G_{c}$.

(3) $$\omega_{close}\ll \omega_{low}$$

(4) $$\omega_{inhibit}\gg \omega_{high}$$

**Figure 4:**
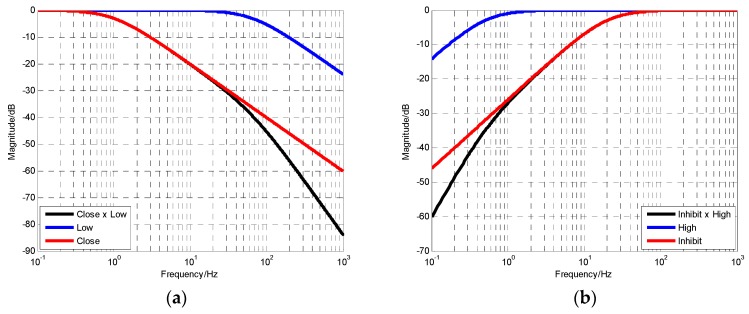
The result of multiplying two transfer functions when cut-off frequency differ greatly: (**a**) Low-pass product results; (**b**) High-pass product results.

Based on Equations (3) and (4), the two approximate transformations can be obtained.

(5) $$G_{close}G_{low}\approx G_{close}$$

(6) $$G_{inhibit}G_{high}\approx G_{inhibit}$$

where $\omega_{low}$, $\omega_{high}$, $\omega_{close}$ and $\omega_{inhibit}$ respectively represent the cutoff frequency of the corresponding transfer characteristics. The numerical simulation of Equations (5) and (6) are shown in Figure 4.

Hence, if the above design requirements have been satisfied, Equation (2) can be approximately reformulated as

(7) $$G_{cl\_fusion}\approx G_{close}+G_{inhibit}\approx 1$$

As a result, the fusion problem is converted into the design problem of CLF filter. Consider a low-bandwidth sensor as a first-order low-pass filter. A high-bandwidth sensor can be expressed as a first-order high-pass filter.

(8) $$G_{low}(s)=\frac{\omega_{low}}{s+\omega_{low}}$$

(9) $$G_{high}(s)=\frac{s}{s+\omega_{high}}$$

According to the frequency characteristic of $G_{close}$ and $G_{inhibit}$, we can assume that the closed-loop transfer function of the CLF filter is a first-order low-pass filter:

(10) $$G_{close}=\frac{\omega_{c}}{s+\omega_{c}}$$

Then the suppression transfer function of the CLF filter follows that

(11) $$G_{inhibit}=\frac{s}{s+\omega_{c}}$$

Thus, the transfer function of CLF is ultimately given by

(12) $$G_{cl\_fusion}=\frac{\omega_{c}}{s+\omega_{c}}\cdot \frac{\omega_{low}}{s+\omega_{low}}+\frac{s}{s+\omega_{c}}\cdot \frac{s}{s+\omega_{high}}$$

In order to achieve the optimal fusion effect, the deviation of $\left|G_{cl\_fusion}\right|$ and 1 should be minimized in the desired frequency domain.

According to Equations (3) and (4), we can get

(13) $$\omega_{high}<\omega_{c}<\omega_{low}$$

Let k be the fusion ratio, and the value of $\omega_{c}$ can be expressed as

(14) $$\omega_{c}=\omega_{low}\cdot k+\omega_{high}\cdot (1-k)k\in [0,1]$$

When k=0, we can get $\omega_{c}=\omega_{high}$, the same can be achieved; if k=1, then $\omega_{c}=\omega_{low}$.

**Figure 5:**
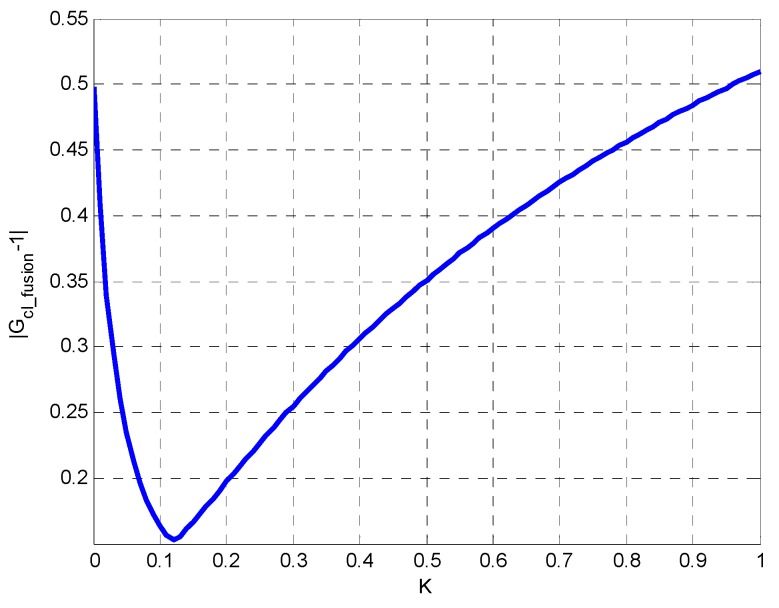
The fusion error of $|G_{cl\_fusion}-1|$ when k is different.

Figure 4 shows the errors between the fusion output performance and the desired performance when the fusion ratio k is different. The simulation conditions are $\omega_{low}=85\times 2\pi \;(\mathrm{rad}/s)$ and $\omega_{high}=1.6\times 2\pi \;(\mathrm{rad}/s)$. The closed-loop characteristics of filter is designed as a first-order low-pass filter. It can be seen that the errors of the fusion output are the smallest when k=0.06, from Figure 5. Therefore, k=0.06 is the optimal fusion ratio. The corresponding cut-off frequency of fusion filter $\omega_{c}$ is 5.196×2π (rad/s).

We can find that fusion results are related to the transfer function of $G_{close}$ through the above simulation example. In order to analyze the influence of filter order on the fusion effect, the closed-loop characteristic of fusion filter is designed as a second-order or even a third-order low-pass expression. Assuming that the second-order low-pass expression is given by

(15) $$G_{close}={\left(\frac{\omega_{c}}{s+\omega_{c}}\right)}^{2}$$

The third-order low-pass expression can be expressed as

(16) $$G_{close}={\left(\frac{\omega_{c}}{s+\omega_{c}}\right)}^{3}$$

According to aforementioned design steps, the different order $G_{close}$ is used to achieve closed-loop fusion, and different fusion error results are obtained. The second-order low-pass expression is corresponding to the optimal fusion ratio that k=0.23. k=0.33 is the optimal parameter of that third-order low-pass filter use.

**Figure 6:**
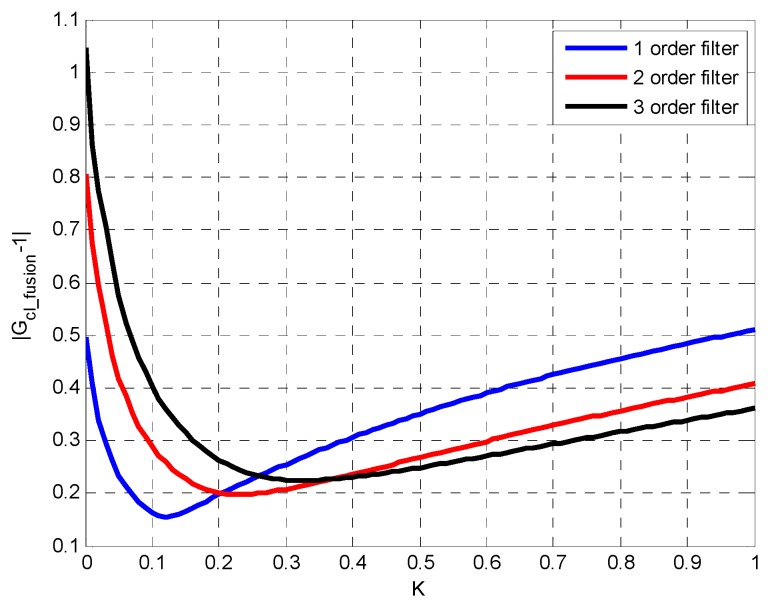
The fusion error of $|G_{cl\_fusion}-1|$ when the filter order is different.

From Figure 6, it can be concluded that the larger the order, the worse fusion precision. Therefore, $G_{close}$ is assumed to be in first-order low-pass form, resulting in best fusion accuracy.

## 3. Inertial Sensors Fusion Experiment

### 3.1. Optical Tracking Experimental Platform

**Figure 7:**
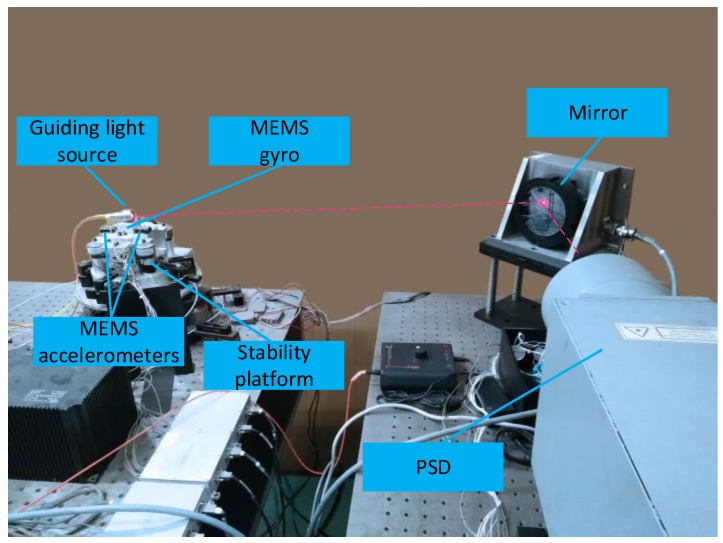
The optical tracking experiment platform.

The inertia sensors fusion experiments were performed to further verify the performance of the proposed optimal design methods. The target tracking experimental platform, as shown in Figure 7, comprised of mirror steering servo system, optical maser, position sensitive detector (PSD), a MEMS gyro, two MEMS accelerometer sensors, and digital processing center. In this experiment, the mirror steering rotated only by one axis, and the optical maser was mounted on rotation axis in order to achieve target tracking. The digital processing center was responsible for the collection and processing of each sensor data in the system, as well as the control algorithms that implement the system. PSD acted as a position detector. MEMS gyro measured the angular velocity of rotation axis and a couple MEMS accelerometers were used to obtain angular acceleration. Assume that accelerations of two MEMS accelerometers can be expressed as $a_{1}(t)$ and $a_{2}(t)$, respectively. L represents the distance between two accelerometers. The angular acceleration is solved by the following expression.

(17) $$\overset{\cdot \cdot }{\theta }(t)=(a_{2}(t)-a_{1}(t))/L$$

In order to obtain better controlled object characteristics, and improve the robustness and stabilization of the system, MEMS gyro was used to realize the velocity inner loop. Due to the resonance of the gyro sensor itself and the limitation of the measurement range, it is necessary to design a corresponding notch filter, and use MEMS accelerometer to assist the acquisition of high frequency signals to realize the measurement bandwidth of the extended gyro. Throughout the experiment, the frequency responses of the platform and sensor were measured by a frequency response meter. Among them, the frequency characteristics of the accelerometer and the gyro given in the paper were obtained by dividing the two signals by using the PSD sensor with absolute position offset as the reference signal.

### 3.2. Transfer Function of MEMS Gyro and Compensation Technique

**Figure 8:**
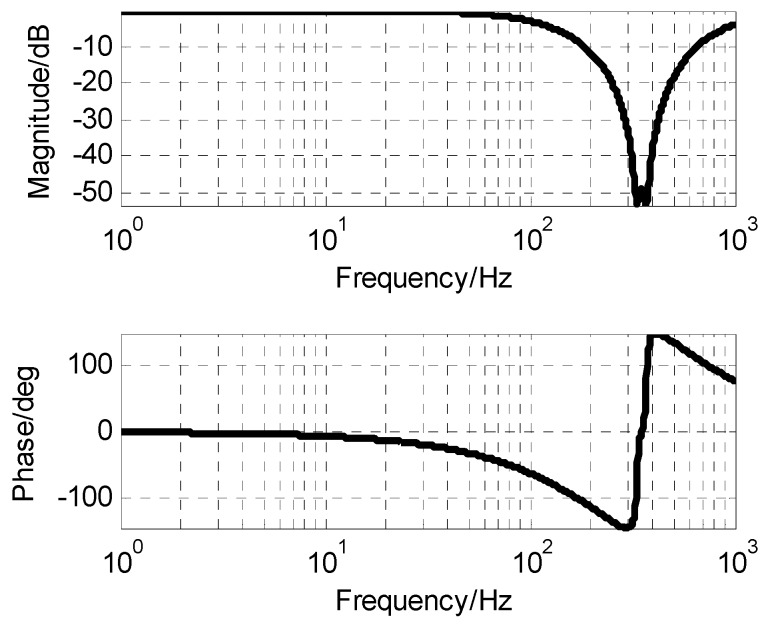
The frequency characteristic of notch filter.

Due to the adopted closed-loop fusion method, the requirement of high frequency response capability of MEMS gyro will be lower. Thus, the notch filter with better attenuation performance can be applied to eliminate resonance effects of MEMS gyro. The notch filter was designed as the following form. As seen in Figure 8, the notch filter can provide attenuation ability beyond −50 dB to resonance of MEMS gyro.

(18) $$G_{Notch}(s)=\frac{\left[{\left(4.3e-4s\right)}^{2}+1.6e-5s+1\right]}{\left[{\left(4.8e-4s\right)}^{2}+1.9e-5s+1\right]}\cdot \frac{\left[{\left(5e-4s\right)}^{2}+1e-3s+1\right]}{\left[{\left(4e-4s\right)}^{2}+8e-4s+1\right]}$$

**Figure 9:**
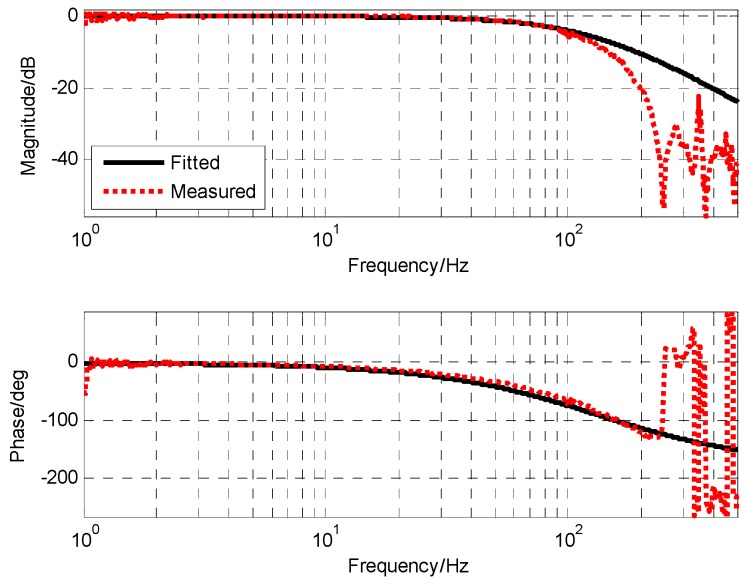
The frequency characteristic of filtered MEMS gyro (PSD as reference sensor).

Figure 9 shows the frequency characteristics of MEMS gyro after adding the notch filter. The resonance of MEMS gyro was well-suppressed and the amplitude was reduced below −20 dB. Furthermore, its transfer function was obtained by fitting.

(19) $$G_{MEMS\_Gyro}(s)=\frac{1}{{\left(0.0012s+1\right)}^{2}}$$

### 3.3. Transfer Function of MEMS Accelerometers and Compensation Technique

**Figure 10:**
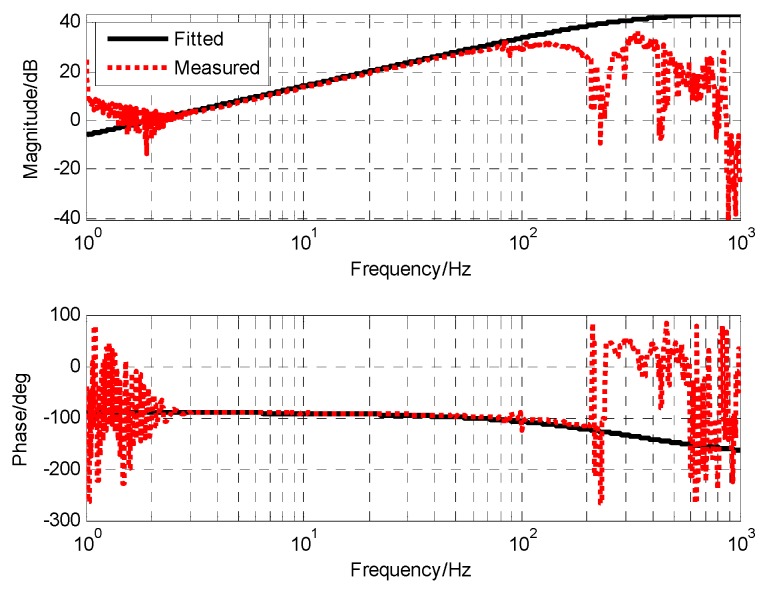
The frequency characteristic of MEMS accelerometers (PSD as reference sensor, one integration).

The frequency response of MEMS accelerometers is shown as Figure 10, and compensates one integration. That is, relative to angular velocity, the difference between the two is a differential, so the transfer function of the accelerometer is

(20) $$G_{MEMS\_Acc}(s)=\frac{s}{\left(5.1e-4s+1\right)}$$

In order to process the acceleration into a velocity signal, the simplest method is to directly integrate the acceleration. However, in practice, the low frequency signal-to-noise ratio of the acceleration is very low, which leads to serious saturation and drift problems. Considering that only the high frequency part of the signal is measured using an accelerometer, the acceleration signal may not be integrated, but only a filtering step is added to change the characteristics of the accelerometer.

(21) $$G_{MEMS\_Acc}(s)=\frac{s}{\left(Ts+1)(5.1e-4s+1\right)}$$

In the selection of the filter coefficient T, if T is too small, it is not conducive to the suppression of high frequency noise of accelerometers. If T is too large, it will affect the characteristics after fusion. In combination with the characteristics of the gyro, the filter coefficient T of the accelerometer is selected to be 0.1 s, so that the transfer function of the accelerometer is

(22) $$G_{MEMS\_Acc}(s)=\frac{s}{\left(0.1s+1)(5.1e-4s+1\right)}$$

**Figure 11:**
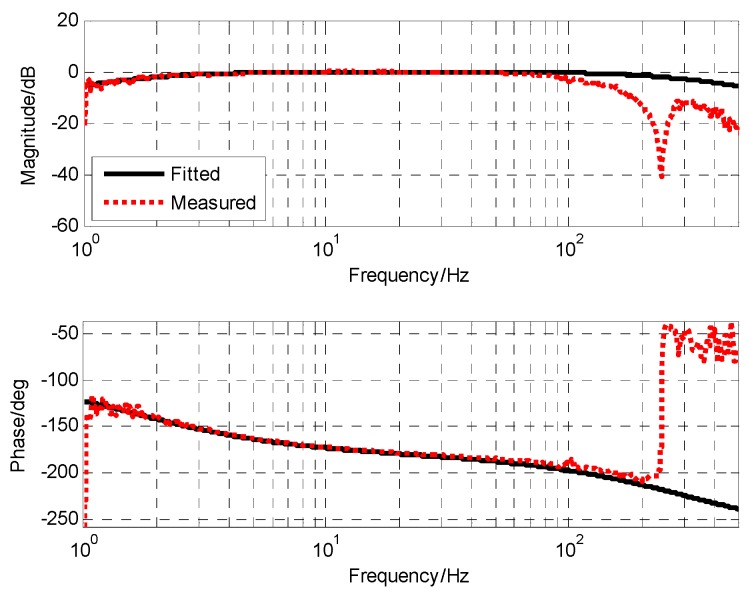
The open-loop Bode with MEMS gyroscope.

After the modification of the filtering link, the accelerometer had similar characteristics to the angular rate sensor and the angular displacement sensor. Its frequency characteristics were similar to a high-pass filter, which can make good use of the accelerometer’s high-frequency response capability and reduce its output noise. Figure 11 shows the frequency characteristic of the accelerometer after filtering. The frequency point of −3 dB was 1.6 Hz. However, in the high frequency part, the mechanical resonance of the test bench had an influence on the measurement.

### 3.4. Closed-loop Fusion Experiment of MEMS Gyro and MEMS Accelerometers

**Figure 12:**
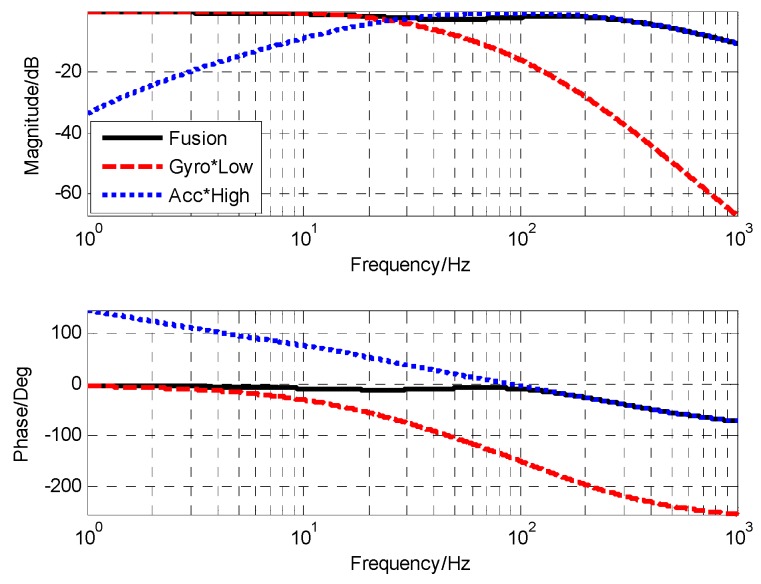
The simulation result of closed-loop fusion.

**Figure 13:**
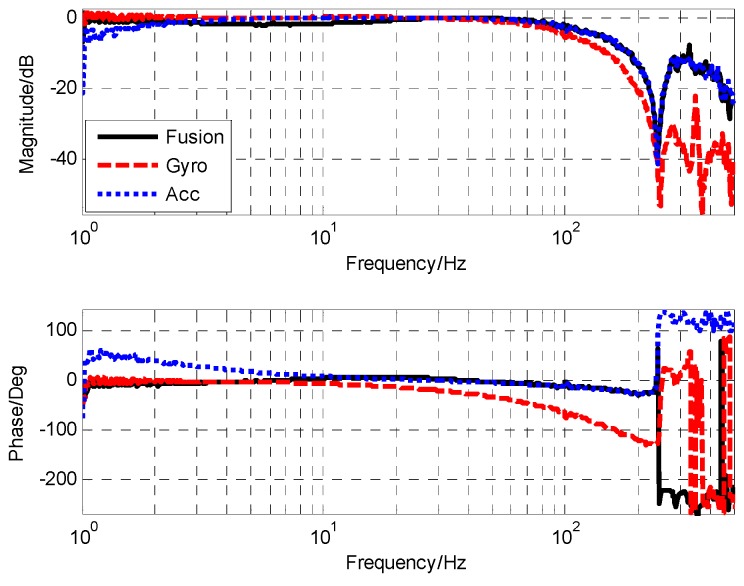
The experiment result of closed-loop fusion.

After adding notch filter, the corresponding cut-off frequency point of gyro was 85 Hz, and accelerometer was 1.6 Hz. When the closed-loop form of the closed-loop fusion filter was designed as the first-order filtering characteristic, the value of k was 0.29 and $\omega_{c}$ was 162.1. The corresponding simulation and experimental results are shown in Figure 12 and Figure 13, respectively.

From the measured fusion results in Figure 13, it can be seen that the experimental results were basically consistent with the simulation results. In the low frequency part, the fusion characteristic coincided with the gyro. In the high frequency part, the fusion characteristic coincided with the accelerometer. The maximum amplitude attenuation was −2.1 dB and the corresponding phase lag was 6.6.

### 3.5. Velocity Closed-Loop Control Experiment Based on Fusion signal

**Figure 14:**
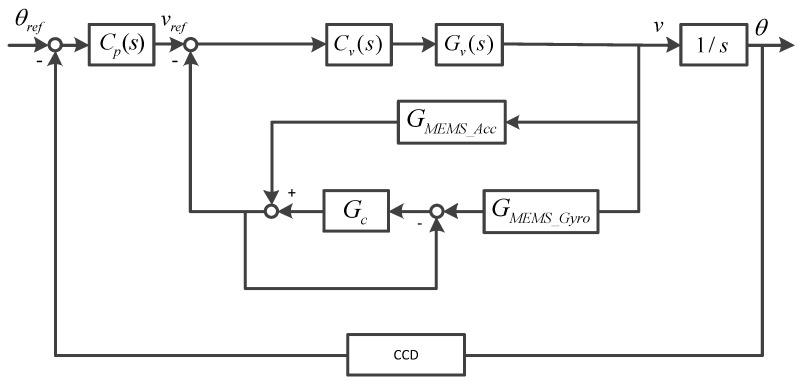
The control structure of OTT.

Figure 14 shows the schematic diagram of the dual closed-loop control of OTT based on closed-loop fusion. The method of using the combining filter requires an accurate sensor prior model, which may be difficult to obtain in practical engineering, and the Kalman filter based method faces the problem of difficulty in establishing a high-order Kalman model. Therefore, we used the closed-loop fusion method based on optimal filter design proposed in this paper to expand the measurement range of the sensor. It is important to emphasize that drift and noise of inertial sensors are two unavoidable problems. However, in the servo control based on the OTT system, a double closed-loop control strategy was adopted. Which were:

(1) Use the gyro to realize the inertial stability loop of the system to improve the stability of the system.
(2) The position detector realizes the position tracking loop to ensure the tracking performance of the system.

**Figure 15:**
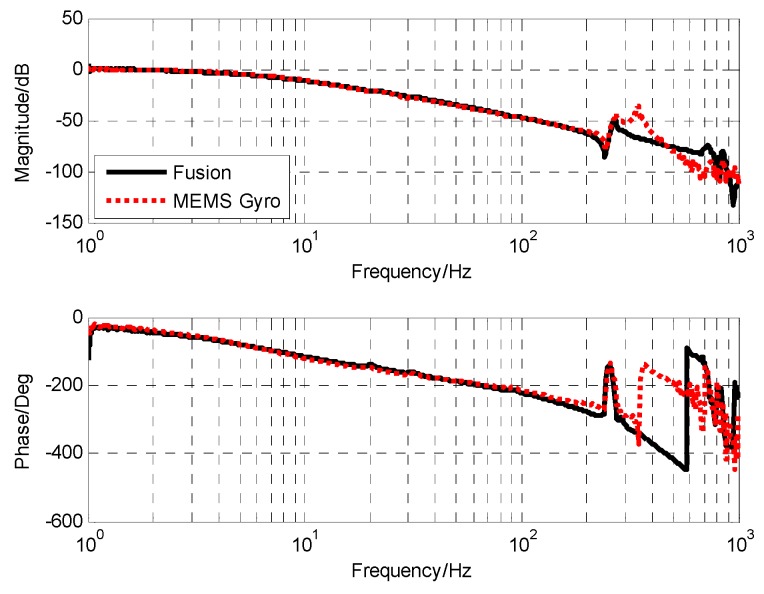
The platform characteristics.

In such a control strategy, the drift problem of the gyro can be effectively compensated by the position loop. In the controller design, the corresponding filter can also be added to suppress the corresponding noise. Therefore, the influence of sensor drift and noise on OTT control is basically negligible, and we can expand the measurement range of low-bandwidth sensors. The characteristics of the platform in Figure 15 were measured using the fusion data. Compared with the characteristics of the gyro measurement, the characteristics of the two were basically the same in the frequency range of 0 to 100 Hz. However, at high frequency, the characteristics of the fused gyro were not affected by the resonance of 350 Hz gyro, so the amplitude attenuation and phase lag caused by the resonance link could be reduced and the correction bandwidth could be improved.

**Figure 16:**
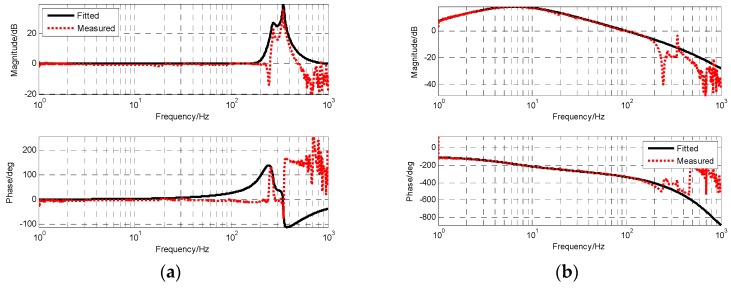
The frequency characteristics of OTT based on MEMS gyro: (**a**) The resonance response of platform; (**b**) the open-loop frequency response of platform.

**Figure 17:**
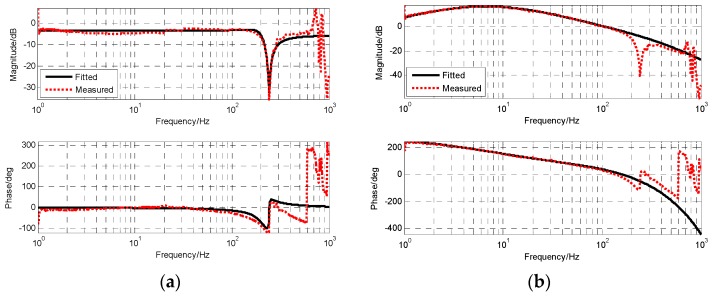
The frequency characteristics of OTT based on closed-loop fusion with optimal design: (**a**) The resonance response of platform; (**b**) The open-loop frequency response of platform.

As can be seen from Figure 16a and Figure 17a, when there is no influence of MEMS gyro resonance, the mechanical resonance of the platform can be eliminated by using a single notch filter. We can clearly see that the frequency characteristics of the sensor measured based on the closed-loop fusion method are more regular and smooth, which reduces the influence of sensor characteristics on the measurement of the frequency characteristics of the object. From the results of open-loop frequency response in Figure 16a and Figure 17a, a better controlled OTT characteristic can be obtained when closed-loop fusion with optimal design is used, which also means that better closed-loop control can be achieved.

According to the fitting result in Figure 17b, the transfer function of stability platform is given as

(23) $$G_{vel\_object}(s)=0.2725\frac{s}{\left(0.03031^{2}s^{2}+0.04917s+1)(0.000037s+1\right)}e^{-1.2e-3}$$

Compared with the characteristics of the stability platform measured directly by MEMS gyro, the lag time of the pure lag link of the fusion scheme is reduced from 1.45 × 10^−3^ to 1.2 × 10^−3^ s, and the stability margin can be increased. More advantageous is that the stable platform characteristic of the fusion scheme completely eliminates the influence of gyro resonance, and the amplitude margin can be guaranteed even after the control bandwidth is increased.

Thus, the controller of velocity loop is designed as

(24) $$G_{vel\_correct}(s)=2.76e+6\frac{\left(0.03031^{2}s^{2}+0.04917s+1)(0.011s+1\right)}{s^{2}(16s+1)(0.00037s+1)}$$

Furthermore, the open-loop transfer function is obtained by

(25) $$G_{vel\_correct}(s)=7.52e+5\frac{\left(0.011s+1\right)}{s(16s+1){\left(0.000037s+1\right)}^{2}}$$

**Figure 18:**
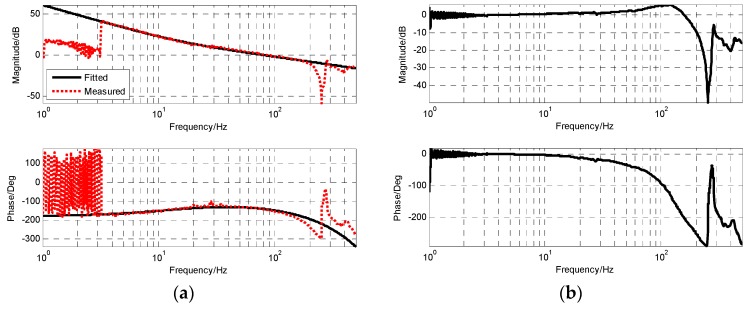
The system control result based on closed-loop fusion with optimal design: (**a**) The frequency response of open-loop; (**b**) the frequency response of closed-loop.

**Figure 19:**
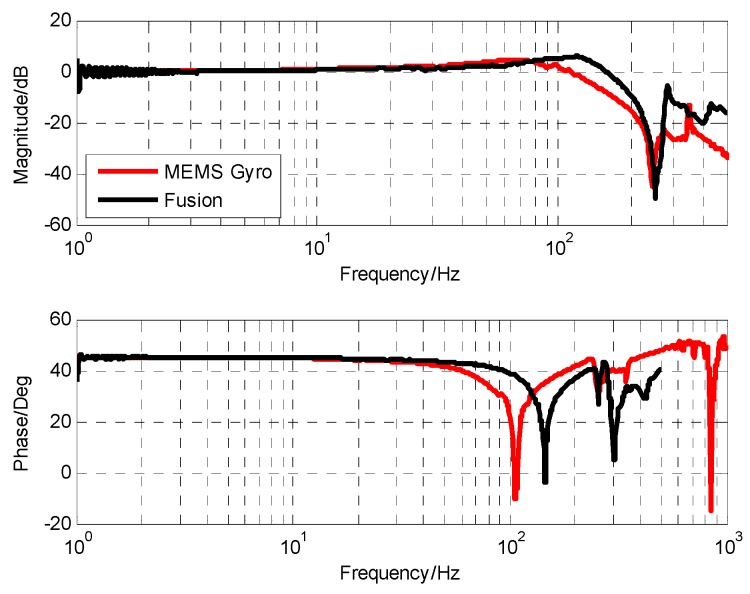
Closed-loop result comparison diagram.

Figure 18a,b shows the open-loop characteristics and closed-loop characteristics of the gyro stability loop, respectively. The open-loop shear frequency is 80.3 Hz and the phase margin is 40 degrees. A high open-loop cutoff frequency means a high closed-loop bandwidth under the conditions that satisfy the open-loop stable phase margin. The final closed loop bandwidth is approximately 172 Hz. The final closed-loop comparison experiment (Figure 19) also verifies that the closed-loop bandwidth based on the closed-loop fusion method with optimal design is about 44.3 Hz higher than that of MEMS gyro. High-speed closed-loop bandwidth further increases the stability of the system, which is in line with our expectations.

## 4. Conclusions

In this paper, the elimination of MEMS gyro resonance and the expansion of measurement range are realized by CLF. CLF is a high-performance fusion method for multi-sensor fusion technology that does not require accurate estimation of the sensor’s transfer function and noise model. However, so far, there are almost no literature to analyze the design of its filters. Therefore, based on the control theory, we proposed the optimal design of the CLF controller. As shown, the controller design algorithm has been proven to have a high degree of fusion performance and the ability to effectively eliminate frequent joint point fusion errors. Finally, the velocity fusion experiment of MEMS gyro and MEMS accelerometers were utilized to expand the velocity loop bandwidth based on optical tracking platform. Simulation and experimental results show that the fusion accuracy is satisfactory and can achieve higher velocity closed-loop bandwidth. Closed-loop fusion can expand the measurement range of the sensor to achieve the purpose of increasing the closed-loop bandwidth. However, from the mathematical principle of closed-loop fusion, it is impossible to compensate for the direct-current drifting and a random-walk effect of inertia sensors. Therefore, the problem of sensor drift correction based on closed-loop fusion architecture in the future is worthy of further study.
